# Seeing the invisible: Reorienting cardiovascular risk prediction across the disease trajectory

**DOI:** 10.1371/journal.pmed.1005263

**Published:** 2026-10-21

**Authors:** Xun Tang, Pei Gao

**Affiliations:** 1 Institute of Advanced Clinical Medicine, Peking University, Beijing, China; 2 Department of Epidemiology and Biostatistics, School of Public Health, Peking University, Beijing, China; 3 Key Laboratory of Epidemiology of Major Diseases (Peking University), Ministry of Education, Beijing, China

## Abstract

In this Perspective, Xun Tang and Pei Gao discuss how cardiovascular risk prediction is simultaneously advancing on two axes: broadening predictors toward molecular omics, and expanding outcomes toward earlier subclinical atherosclerosis and later heart failure.

## Teaser

Current risk equations miss many cardiovascular events in people classified as low risk. Prediction is simultaneously advancing on two axes: broadening predictors toward molecular omics, and expanding outcomes toward earlier subclinical atherosclerosis and later heart failure.

## Introduction

For decades, cardiovascular risk prediction has relied on a series of foundational algorithms, evolving from the Framingham model and Pooled Cohort Equations to the contemporary PREVENT, SCORE2, QRISK4, PREDICT, and China-PAR models, etc. [1]. All are multivariable equations, fitted by survival regression to large cohorts, that estimate an individual’s absolute cardiovascular risk (usually 5–30 years) from demographic and traditional clinical factors such as age, sex, blood pressure, cholesterol, smoking, and diabetes. These tools have been widely adopted in clinical practice and have substantially improved risk stratification and preventive care worldwide. However, despite their demonstrated utility, they still rest on an uncomfortable fact: a large share of events occurs in people whom our current equations judge to be at low risk. In the Prospective Urban Rural Epidemiology (PURE) study, 41% of first major cardiovascular events occurred in individuals with a low predicted risk, defined by the estimated 10-year World Health Organization risk [2]. Given that preventive therapies like statins are typically targeted according to predicted risk, these apparent low-risk individuals may be systematically excluded from interventions that could otherwise alter their disease trajectory, underscoring the real-world consequences of residual risk misclassification. This “low-risk paradox” points to a key limitation: equations built solely on traditional cardiovascular factors fail to measure a meaningful component of residual risk.

One approach to improve prediction is to expand the range of predictors. However, after decades of research, within the cardiovascular–kidney–metabolic framework, individual clinical measures now often add only a marginal improvement to the C-index (a measure of discrimination of how well a model separates people who will develop the event from those who will not, ranging from 0.5, no better than chance, to 1.0, perfect), typically less than 0.01 [3]. We argue that escaping this ceiling may require moving along two axes simultaneously: what is measured and what is predicted, as shown in Fig 1. Together, these define a new landscape for cardiovascular risk prediction.

**Fig 1:**
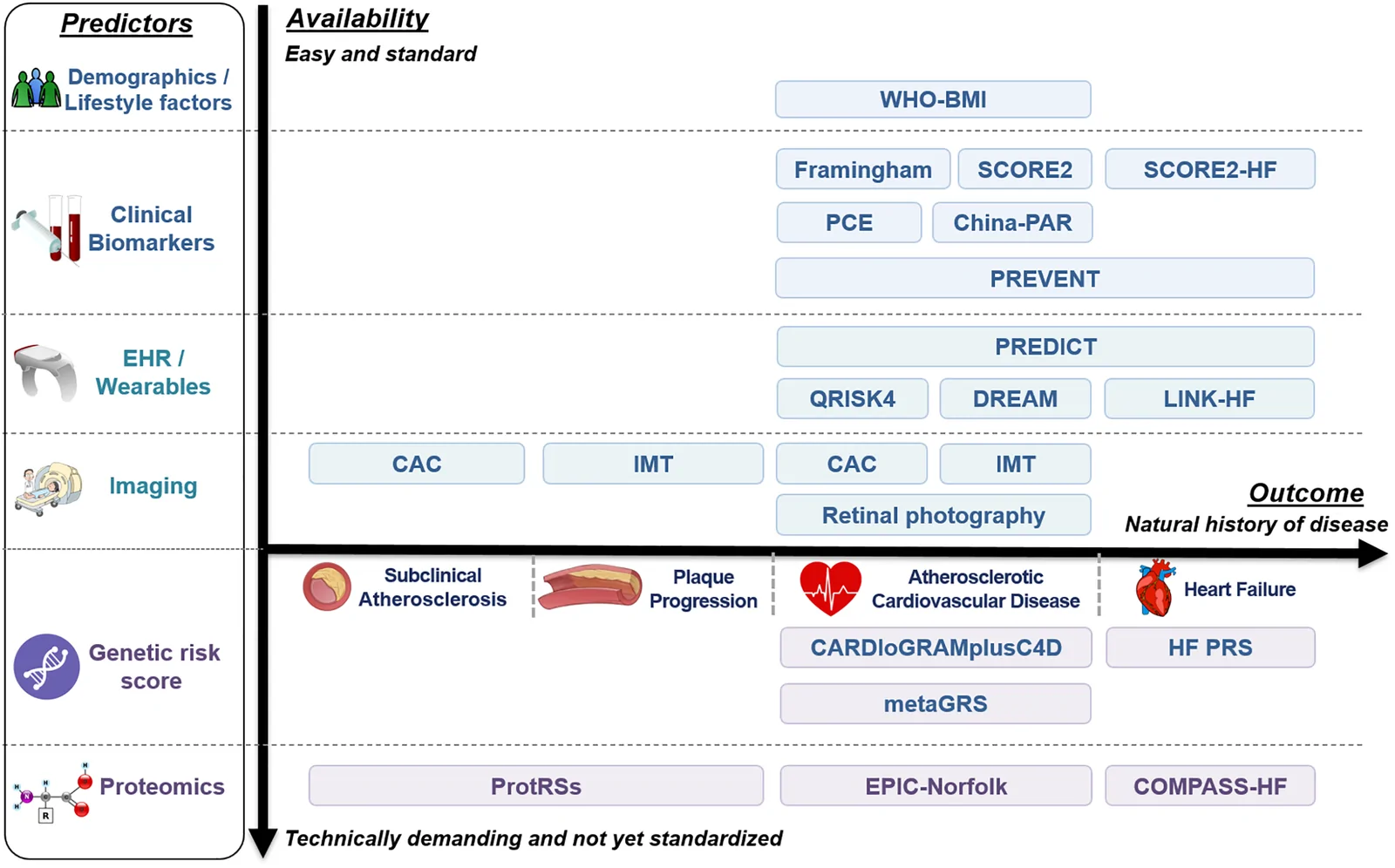
Established and emerging cardiovascular risk prediction tools positioned along two axes of reorientation. Risk prediction is advancing along two dimensions. The vertical axis orders the predictors built into a model by their availability, from the most accessible demographic and lifestyle factors and clinical biomarkers, through data from electronic health records and wearable devices, and imaging, down to the molecular genetic and proteomic measures, which remain technically demanding and not yet standardized. For each risk prediction tool, its position on this axis reflects the most difficult-to-measure predictor it incorporates, not the full set of predictors used. The horizontal axis orders the outcome predicted along the natural history of disease, from subclinical atherosclerosis to heart failure. Representative tools are placed according to the predictors they use and the outcome they target. Labels often refer to risk prediction models when named models exist. For models without a formal name, the label identifies the study that developed the tool. Models built on routinely collected electronic health record or wearable data include QRISK4 and PREDICT; the DREAM score, which repeatedly updates short-term cardiovascular risk from real-world EHR data to flag impending atherosclerotic cardiovascular disease; and LINK-HF, in which continuous wearable monitoring analytics predicted heart failure hospitalization. For imaging-based approaches, labels denote the imaging type rather than a specific model or study. The protein risk scores (ProtRSs) discussed in this Perspective pair a proteomic predictor with an early, subclinical target (lower left), a frontier that established clinical scores do not reach. *ASCVD, atherosclerotic cardiovascular disease; BMI, body mass index; CAC, coronary artery calcium; EHR, electronic health record; GRS, genetic risk score; HF, heart failure; IMT, intima-media thickness; PCE, Pooled Cohort Equations; PRS, polygenic risk score; ProtRS, protein risk score; WHO, World Health Organization. Icons from Openclipart (CC0) and Wikimedia Commons (CC0 and NIH/NHLBI public domain).*

## Two axes of reorientation

In Fig 1, the vertical axis (the predictors) is now being reshaped by the omics revolution. Genome-wide polygenic risk scores reveal lifelong susceptibility and are well suited to early-life stratification and primordial prevention; but because genotypes are fixed at conception, they cannot capture the dynamic state of disease. The plasma proteome offers a complementary, fundamentally different signal, capturing the real-time interplay of genome, environment, and disease activity [3]; unlike genotype, it is dynamic and potentially modifiable, so it could, in principle, track both baseline risk and the response to intervention. Falling assay costs and high-throughput platforms now let us measure hundreds to thousands of proteins from a single blood draw [4,5], bringing proteomic scores within reach of large-scale use. Targeted panels of myocardial stress, inflammation, and metabolic proteins have already been linked to incident cardiovascular events [6,7], though in large studies they have added only modest discrimination to conventional scores [4,5]. Alongside the molecular and imaging predictors on this axis, dynamic monitoring is a further, complementary category: wearable devices capture continuous, real-world physiology between clinic visits, letting risk be estimated dynamically and repeatedly rather than from a single static measurement. Such signals gain real-world value when fed into electronic health record (EHR) systems that can act on them; this is feasible at scale, as the DREAM score shows by repeatedly updating short-term cardiovascular risk from real-world EHR data to trigger automated early warnings [8], while on the wearable side LINK-HF showed that continuous monitoring analytics predicted heart failure hospitalization [9].

The horizontal axis in Fig 1 (the outcomes) is expanding in both directions along the natural history of disease. At the earlier end, the target is shifting from clinical events to subclinical atherosclerosis, which is common even at low conventional risk [10] and whose progression, integrating cumulative exposures with individual susceptibility, independently forecasts future events [10,11]. Coronary artery calcium (CAC) scoring already shows that detecting subclinical disease can reclassify risk and guide statin allocation, but it depends on imaging that is neither cheap nor universally available [11]. At the later end, newer algorithms have begun to incorporate heart failure as a distinct outcome, reflecting its rising burden and the potential for prevention even in forms such as heart failure with preserved ejection fraction. Accordingly, PREVENT now estimates heart failure risk alongside atherosclerotic cardiovascular disease (ASCVD), and SCORE2-HF extends the original SCORE2 for the outcome of heart failure to the general population [12].

Together, the two axes reframe cardiovascular risk prediction as managing a trajectory rather than forecasting a single event. Exemplifying this, a recent study in *PLOS Medicine* [13] occupies the intersection of the two axes (Fig 1, lower left), pairing an omics predictor with an early outcome by linking the plasma proteome to the progression of subclinical atherosclerosis. From repeated CAC and carotid plaque imaging in a Chinese community cohort, the investigators derived protein risk scores (ProtRSs) for each trait, then tested them against incident ASCVD in a separate case-cohort benchmarked to the China-PAR model. A blood-based signature of that progression, as the ProtRSs show, could deliver similar information more accessibly, flagging candidates for intensified prevention before irreversible injury and moving the gateway of prevention forward [11].

## From statistical signal to clinical utility

Realizing this vision, however, requires confronting the distance between a statistical signal and a clinically useful tool. This distance is a general one, not specific to any single study: adding proteins to conventional risk factors has generally improved discrimination only modestly [4,5]. The study by Huang and colleagues [13] is a case in point: its scores raised the C-index by only 0.018 over China-PAR and, on their own, discriminated incident ASCVD a little better than age and sex. Their value lies instead in net reclassification improvement, which also proved double-edged, returning about one in five intermediate-risk future events to low risk. The hurdles it faces are shared across the field. Scores derived and validated within a single population and platform must still prove themselves across ancestries and assays, and those trained to predict an imaging endpoint must also show value over directly measuring that endpoint. Meeting these tests will determine whether molecular risk scores earn a place in prevention. The repeated-measures design, internally consistent signatures, and acceptable calibration demonstrated in this study [13] are the right foundation on which to build.

In summary, cardiovascular risk prediction is reorienting: predictors are broadening from clinical factors to molecular signatures, while outcomes along the natural history of disease are expanding earlier to subclinical atherosclerosis and later to heart failure. The proteomic signatures examined here lie at the current frontier of cardiovascular risk prediction. They make visible a risk that clinical factors cannot, while stopping short of external validation across populations. The way forward is clear. Evidence that acting on these scores prevents disease cost-effectively is required. That acting on such a signal can change outcomes is no longer hypothetical: in a pragmatic randomized trial, an AI-enabled electrocardiogram alert that flagged hospitalized patients at high risk of death prompted more timely intensive care and significantly lowered 90-day all-cause mortality [14]. Longitudinal designs that test whether within-person change tracks treatment response are also needed. Translation can also flow backward, as population-derived signatures, here pointing to cathepsin D and IL-2RA, suggest mechanisms and drug targets for the bench. In addition, machine-learning methods that extend regression-based models can help, pushing prevention toward a more proactive, personalized paradigm. The omics era will not abolish clinical risk scores; it will be judged by whether it extends their reach to the many patients those scores now overlook.

## Abbreviations

CAC: Coronary artery calcium
EHR: electronic health record
PURE: Prospective Urban Rural Epidemiology
